# Impact of a Transition Clinic on Long-Term Care and Nutritional Management in Patients with Inborn Errors of Metabolism

**DOI:** 10.3390/nu17203240

**Published:** 2025-10-15

**Authors:** Everardo Josué Díaz-López, Antia Fernández-Pombo, Álvaro Hermida-Ameijeiras, Eva Gómez-Vázquez, Gemma Rodríguez-Carnero, Noemí Jiménez-López, Rocío Villar-Taibo, Ana Cantón-Blanco, Virginia Muñoz-Leira, Paula Sánchez-Pintos, Maria-Luz Couce, Miguel A. Martínez Olmos

**Affiliations:** 1 Division of Endocrinology and Nutrition, University Clinical Hospital of Santiago de Compostela, 15706 Santiago de Compostela, Spain; antiafpombo@gmail.com (A.F.-P.); eva.gv_83@hotmail.com (E.G.-V.); mrcarnero@alumni.unav.es (G.R.-C.); rotaibo22@gmail.com (R.V.-T.); ana.canton.blanco@sergas.es (A.C.-B.); virginia.munoz.leira@sergas.es (V.M.-L.); 2 Unidade de Enfermedades Tiroideas e Metabólicas (UETeM)-Molecular Pathology Group, Department of Psychiatry, Radiology, Public Health, Nursing and Medicine, Center for Research in Molecular Medicine and Chronic Diseases (CIMUS), University of Santiago de Compostela, 15782 Santiago de Compostela, Spain; 3 Division of Internal Medicine, University Clinical Hospital of Santiago de Compostela, 15706 Santiago de Compostela, Spain; alvaro.hermida@usc.es; 4 Unit for Diagnosis and Treatment of Congenital Metabolic Disorders, University Clinical Hospital of Santiago de Compostela, Health Research Institute of Santiago de Compostela (IDIS), European Reference Network for Hereditary Metabolic Disorders (MetabERN), 15706 Santiago de Compostela, Spain; paula.sanchez.pintos@sergas.es; 5 Division of Endocrinology and Nutrition, Hospital Universitario 12 de Octubre, 28041 Madrid, Spain; noemijl815@gmail.com; 6 Division of Pediatrics, University Clinical Hospital of Santiago de Compostela, Spanish Network in Maternal, Neonatal, Child and Developmental Health Research (RICORS-SAMID), Center for Biomedical Network Research (CIBERER), 15706 Santiago de Compostela, Spain; 7 Molecular Endocrinology Group-Health Research Institute of Santiago de Compostela-IDIS, 15706 A Coruña, Spain; 8 CIBER of Physiopathology of Obesity and Nutrition (CIBEROBN), Carlos III Health Institute, 28029 Madrid, Spain

**Keywords:** inborn errors of metabolism, transition to adult care, metabolic disorders, dietary adherence

## Abstract

**Background/Objectives**: The transition from pediatric to adult care in inborn errors of metabolism (IEM) is considered important to ensure continuity of care, adherence to treatment, and long-term metabolic control. However, transition processes are often delayed, and standardized protocols are lacking, which can negatively impact patient outcomes. This study aimed to evaluate the impact of structured transition consultations on adult care engagement, nutritional management, and follow-up adherence in patients with IEM. **Methods**: This retrospective study included 160 patients (59.4% women) diagnosed with IEM and with a mean age of 36.2 ± 11.6 years. Patients were divided into two groups: those who underwent a structured transition consultation (*n* = 41) and those who did not (*n* = 119). Data on demographic and clinical characteristics, dietary management, and follow-up adherence were collected. **Results**: Patients who underwent structured transition consultations were significantly younger at diagnosis (1 [IQR 131] months vs. 66 [IQR 359] months, *p* = 0.001) and at their first adult visit (24.4 ± 9.5 vs. 32.3 ± 10.6 years, *p* < 0.001) compared to those who did not. Neonatal screening (45% of the overall cohort) was more common among these patients (65.9% vs. 37.8%, *p* = 0.007) suggesting a trend toward smoother integration into adult care. The absence of dietary records was considerably more frequent in the non-transition group (43.7% vs. 17.1%), with a significant crude association (*p* = 0.007) that was attenuated after age adjustment (*p* = 0.064). Overall follow-up adherence was high (88.1%) and comparable between groups. **Conclusions**: Structured transition consultations in patients with IEM were associated with earlier participation in adult care, better maintenance of dietary records, and high overall follow-up adherence, even among younger patients typically at higher risk of disengagement.

## 1. Introduction

Inborn errors of metabolism (IEM) are biochemical abnormalities resulting from specific genetic defects that alter the structure or function of proteins or result in their complete absence [1]. To date, more than 1900 IEM have been described (http://www.iembase.org/) [2]. Advances in diagnosis and treatment have markedly improved the prognosis for these conditions, enabling an increasing number of IEM patients to survive into adulthood [3,4]. In this evolving scenario, maintaining metabolic stability remains challenging, with nutritional management as a central element of care. Personalized dietary regimes, often supplemented with specific nutrients, are the cornerstone of current guidelines and, for many IEM, the only effective therapy [5]. However, during adolescence, clinical follow-up often declines and adherence to medical nutritional therapy weakens, leading to metabolic imbalances [6,7,8,9].

The transition consultation for IEM patients has been proposed as a key strategy to maintain continuity of care as they move from pediatric to adult healthcare services [10,11]. Early patient engagement prior to the transition phase can enhance the overall experience and reduce treatment discontinuation [10,12]. A well-structured transition process, tailored to the physical, emotional, and social needs of each individual, is regarded as important to promote long-term adherence and ensure ongoing clinical stability—ideally occurring during a period of remission without major therapeutic changes [1]. Importantly, evidence from specific IEM populations suggests that structured transition incorporating continuous dietary counseling may improve nutritional adherence, a critical determinant of metabolic control in adulthood [13]. Although there is no universally established age threshold, transition is generally recommended between 16 and 18 years [14]. The American Academy of Pediatrics advocates for a structured transition between 18 and 21 years [1], whereas European guidelines recommend early preparation—starting as early as 12–13 years—depending on individual circumstances [15,16].

Building on these recommendations, several institutions have developed dedicated adult outpatient clinics and coordinated transition programs [4,12,17,18]. The Unit for Diagnosis and Treatment of Congenital Metabolic Disorders, located in the University Clinical Hospital of Santiago de Compostela, serves as a Reference Unit for Rare Metabolic Diseases in the Autonomous Community of Galicia, Spain. Since its inception, the Unit has evolved continuously to meet growing care demands and uphold high-quality processes. In January 2015, it was designated a Centre, Service and Unit of Reference (CSUR) by the Spanish Ministry of Health for the treatment of congenital metabolic diseases in both pediatric and adult populations [19]. The Unit was recognized as a European Expert Center in March 2017 and became a member of the European Reference Network for Hereditary Metabolic Disorders (MetabERN), further consolidating its reputation [20]. Since late 2019, it has implemented structured transition consultations to facilitate the smooth transfer of care from pediatric to adult services, ensuring continuity and sustained management for patients with IEM.

Although increasing attention has been given in recent years to the transition of patients with IEM, previous research in this field has been based on reports limited to small samples and has mainly focused on specific conditions, such as phenylketonuria (PKU), therefore resulting in a lack of studies that encompass the full spectrum of IEM [21,22,23].

The current real-world study aims to evaluate the long-term impact of a structured transition consultation program, uniquely encompassing a wide spectrum of IEM and assessing key outcomes such as care continuity, adherence to treatment and nutritional recommendations, and other factors influencing successful transition to adult services.

## 2. Materials and Methods

### 2.1. Study Design

This retrospective, observational cohort study evaluates the demographic and clinical characteristics of 160 patients with IEM managed in the adult outpatient clinic at the Unit for Diagnosis and Treatment of Congenital Metabolic Disorders of the University Clinical Hospital of Santiago de Compostela, Spain. It assesses the impact of the transition from pediatric to adult care on healthcare engagement and treatment adherence outcomes by comparing patients who underwent a structured transition consultation program with those who did not.

### 2.2. Patient Selection

All patients with IEM who were consecutively attended in the adult outpatient clinic between 2019 and 2023 were included in the analysis. The study population was divided into two groups. The first group (*n* = 41) consisted of patients who followed a structured transition consultation program, receiving transition education and participating in joint pediatric-adult consultations. The second group (*n* = 119) comprised patients who did not receive a formal transition process and/or were diagnosed in adulthood, either through clinical presentation or family screening.

### 2.3. Transition Consultation Protocol

The transition consultation protocol was initially developed based on current consensus recommendations for patients with IEM [1]. Inclusion criteria for the structured transition include patients with a confirmed diagnosis of IEM who are clinically stable, demonstrate basic disease knowledge and have achieved an appropriate degree of psychosocial maturity. Specifically, the transition process established at the Unit consists of three phases. In the initial preparation phase, the pediatric team that has followed the patient since diagnosis gradually begins to involve the patient in the management of their disease and provides detailed information to both the patient and their family regarding the upcoming transfer to adult specialists. This preparation phase includes consultations covering disease-specific knowledge, dietary management, recognition of acute symptoms requiring urgent evaluation, self-management of treatment, and promotion of autonomy. During the transition phase (initiated at a minimum age of 15 years and individualized according to the patient’s developmental stage, disease stability, knowledge, skills, and maturity), the pediatrician introduces the patient to the adult care team through at least one joint transition visit, in which both teams participate together. This multidisciplinary team is composed of two endocrinologists, one internist, one dietitian, and one laboratory nurse. Once the transition is completed, during the first two years of follow-up in the adult outpatient clinic, a designated coordinator ensures that the patient attends scheduled appointments regularly, and reschedules them, if necessary, to ensure continuity of care (evaluation phase).

### 2.4. Clinical Assessment and Data Collection

The classification of IEM was based on the International Classification of Inherited Metabolic Disorders (ICIMD), a hierarchical, group-based framework that organizes all known IEM according to shared biochemical, clinical, and pathophysiological features. This classification system facilitates a comprehensive understanding of the interconnections among various metabolic conditions and can be accessed at http://www.icimd.org/.

Demographic and clinical data were retrospectively retrieved from patients’ electronic medical records. The frequency of clinic visits and specific components of patient care were also recorded. Patients were evaluated for pharmacological treatments specific to their IEM. These treatments included enzyme replacement therapies, cofactor supplementation, substrate reduction therapies, or other targeted interventions aimed at addressing the underlying metabolic defect.

### 2.5. Nutritional Adherence

Nutritional adherence was evaluated by an experienced dietitian (EGV) using patient dietary records documented in Odimet^®^ (https://www.odimet.es, accessed on 3 September 2025), a dietary calculation software specifically developed for IEM by our Unit in 2008 and subjected to regular updates (registration number: 03/2008/924). This platform provides detailed quantification of amino acids, macronutrients, micronutrients, and specialized medicinal foods, and allows patients to add new food products in their own profile and interact with their care team to allow dietary modifications [24].

At each scheduled follow-up, the dietitian thoroughly reviewed and updated the dietary records entered in Odimet^®^, classifying adherence as adequate when the prescribed dietary plan was followed within the established ranges, and as inadequate when deviations were identified. In addition to professional evaluation, the platform also allowed continuous monitoring of patient engagement. For this purpose, two aspects were documented: the individual use of Odimet^®^ at home, reflecting the patient’s active role in registering intake or managing their dietary plan outside the clinic; and the overall adherence status, determined from the integration of dietary records and professional assessment in Odimet^®^.

### 2.6. Statistical Analysis

Statistical analyses were performed using IBM SPSS Statistics for Windows version 22.0. The normality of the data distribution was assessed using the Kolmogorov–Smirnov test. Continuous variables were presented as means with standard deviations or medians with interquartile ranges (IQR), depending on distribution. Categorical variables were expressed as numbers and percentages. For group comparisons, the Mann–Whitney U-test or independent samples *t*-test was employed for continuous variables, and the chi-squared test or Fisher’s exact test for categorical variables. Regression analyses (analysis of covariance [ANCOVA] and multivariable logistic regression models) were performed to assess differences between groups, presenting both unadjusted results and models adjusted for age. Kaplan–Meier curves were generated to assess differences in follow-up between groups, and a Cox proportional hazards regression model was applied to evaluate the effect of transition on the risk of loss of follow-up. Hazard ratios (HR) with 95% confidence intervals (CI) were calculated. A *p*-value of < 0.05 was considered statistically significant.

## 3. Results

### 3.1. Patient Demographics and Diagnostic Pathways

A total of 160 patients were included in the study (59.4% women), with an overall mean age of 36.2 ± 11.5 years. Demographic, clinical, treatment, and follow-up characteristics are summarized in Table 1. IEM were diagnosed through neonatal screening in 45.0% of cases, through clinical suspicion in 28.1%, and via family screening in 26.9%. The mean age at diagnosis was 13.1 ± 16.6 years, while the mean age at the first adult consultation was 30.4 ± 10.9 years.

### 3.2. Inborn Errors of Metabolism Distribution

**Figure 1:**
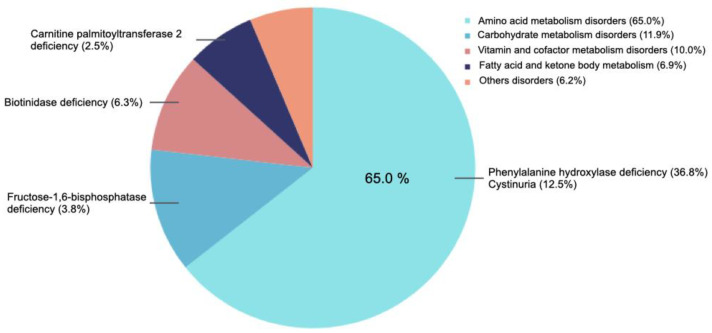
Distribution of inborn errors of metabolism (IEM) in the study population. Phenylalanine hydroxylase deficiency was the most frequent condition, representing more than one-third of cases and underscoring its clinical relevance given the need for lifelong dietary management. Other relatively common disorders included cystinuria and biotinidase deficiency, highlighting the heterogeneity of IEM within the cohort.

In terms of IEM distribution, disorders of amino acid metabolism were the most common (65.0%), followed by carbohydrate metabolism disorders (11.9%). Among the specific conditions, phenylalanine hydroxylase deficiency was the most prevalent (36.9%), with cystinuria (12.5%) and biotinidase deficiency (6.3%) also being common. Less frequent conditions included fructose-1,6-bisphosphatase deficiency and methylenetetrahydrofolate dehydrogenase 1 deficiency, each accounting for 3.8% of cases (Figure 1). No significant differences were found regarding IEM distribution between transition and non-transition groups (*p* = 0.553). The detailed classification and frequency of IEM in the study cohort are presented in Table S1.

### 3.3. Demographic Characteristics of Transitioned Vs. Non-Transitioned Patients

A comparison of the 41 patients who underwent a transition consultation and the 119 who showed no significant difference in sex distribution was carried out (*p* = 0.218). Transitioned patients were significantly younger at their first adult visit (24.4 ± 9.5 vs. 32.3 ± 10.6 years, *p* < 0.001) and at diagnosis (median ≈ 1 month, IQR 131 vs. 66 months, IQR 359; *p* = 0.008). After adjustment for age, no significant differences were observed in the number of visits and follow-up duration, although the first adult visit was significantly earlier in the transition group.

### 3.4. Diagnostic Pathways by Group

Neonatal screening was more frequent in the transition group (65.9% vs. 37.8%), whereas diagnosis through late clinical presentation was more prevalent among patients without transition (32.8%). In addition, a considerable proportion of patients in the non-transition group were diagnosed through family history (29.4%), often following cascade screening after molecular diagnosis in an offspring. The overall distribution of diagnostic pathways differed significantly between groups (*p* = 0.007), although this association was no longer statistically significant after adjustment for age (*p* = 0.259).

### 3.5. Treatment Modalities and Dietary Adherence

Pharmacological treatment was prescribed in 46.3% of patients, with a higher proportion in the transition group (58.5%) compared to the non-transition group (42.0%) after age adjustment (*p* = 0.001). Dietary treatment was recommended to almost all patients (94.4%), without significant differences between groups (100% vs. 92.4%, *p* = 0.064).

Dietary records were available for 63.1% of the cohort, while 36.9% of patients did not provide records during follow-up. The absence of dietary records was considerably more frequent in the non-transition group compared with the transition group (43.7% vs. 17.1%), with a significant crude association (*p* = 0.007) that was attenuated after adjustment for age (*p* = 0.064).

Use of the Odimet^®^ platform by patients themselves was limited (6.9%) and comparable between groups (4.9% vs. 7.6%, *p* = 0.730). Overall, dietary adherence was observed in 51.9% of patients, with no significant differences between the transition (48.8%) and non-transition groups (52.9%, *p* = 0.718).

### 3.6. Follow-Up

**Figure 2:**
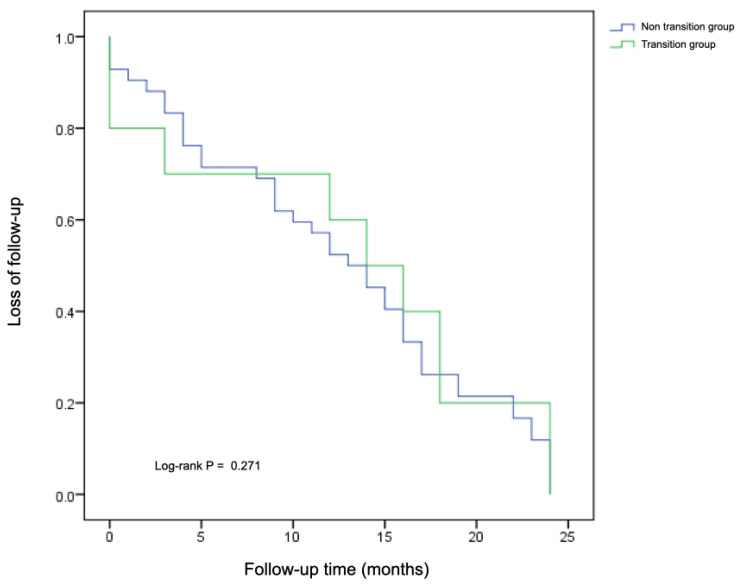
Loss of follow-up over time in the transition group compared with the non-transition group during the two-year post-transition period.

Active follow-up was maintained in 88.1% of patients, while 11.9% discontinued follow-up. A total of 30 patients (25.2%) in the non-transition group and 11 patients (26.8%) in the transition group either missed an appointment or needed to reschedule, yet remained engaged in care. A comparative analysis of appointment attendance revealed no significant differences between groups in the number of scheduled visits (*p* = 0.173), missed appointments (*p* = 0.399), or rescheduled visits (*p* = 0.852). Although the transition group had a higher median follow-up duration (36 months, 95% CI: 31.6–40.4) compared to the non-transition group (29 months, 95% CI: 25.9–32.0) over the two-year post-transition period, this difference was not statistically significant (Log-Rank test, *p* = 0.271). Cox regression analysis confirmed the absence of significant differences, with the transition group showing a HR of 0.83 (95% CI: 0.56–1.23, *p* = 0.348), indicating no increased risk of loss of follow-up compared with the non-transition group (Figure 2).

Discontinuation of care was defined as the event, while patients remaining in active follow-up at the end of the study period were censored.

## 4. Discussion

The transition from pediatric to adult metabolic care is critical for ensuring continuity of care, improving treatment adherence, and achieving better long-term metabolic control [10,25]. In our study, patients undergoing a formal transition process were compared with those who did not.

Notably, the mean age at the first adult visit was significantly lower in the transition group (24.4 ± 9.5 years) than in the non-transition group (32.3 ± 10.6 years). However, the mean transition age of 24 years remains considerably higher than the recommended range of 16–18 years according to international societies for the study of IEM [1,14]. Several factors may account for this delay. Institutionally, structured transition consultations have only recently been implemented in our center, which may partly explain the extended pediatric follow-up. Culturally and psychologically, many patients and their families develop strong and enduring relationships with pediatric teams, often perceiving the transfer to adult care as a disruptive and anxiety-provoking step. In addition, pediatric providers themselves may hesitate to transfer medically complex patients due to concerns about continuity in adult settings. These factors collectively contribute to the postponement of transfer and mirror findings from other contexts. Similar patterns of delayed transition have been documented elsewhere. For example, a study of adults with PKU reported a mean transition age of 31.3 ± 10.4 years, despite early diagnosis in infancy (mean age at diagnosis 7.3 weeks, range 0–260 weeks), highlighting the persistence of prolonged pediatric follow-up into adulthood [22]. Such findings suggest that even structured programs may face challenges in aligning clinical practice with established recommendations, emphasizing the need for continuous system-level efforts to facilitate timely transfer. This delayed transition represents a limitation of the study and underscores the need to implement earlier, guideline-based transition programs.

A key observation in our study is that 45% of patients were diagnosed through neonatal screening, while the remaining 55% were diagnosed later in life. As anticipated, early identification was associated with a younger age at both diagnosis and the first adult clinic visit, highlighting the role of newborn screening in facilitating smoother transitions into adult care. These results are consistent with a multicenter study of 24 adult metabolic centers, which reported that more than 40% of IEM cases are diagnosed in adulthood [14], also underscoring the persistent challenge of late diagnosis [10]. Although advances in newborn screening and diagnostic technologies have substantially improved early detection, a considerable proportion of patients continue to be identified later in life, with potential implications for long-term outcomes. Importantly, the expansion of newborn screening programs, coupled with progress in diagnostic tools, therapeutic strategies, and overall healthcare delivery, has contributed to the growing population of adult patients—both those diagnosed in childhood who survive into adulthood and those newly diagnosed in adult life.

In terms of treatment, pharmacological therapy was more frequently prescribed in the transition group (58.5%) compared with the non-transition group (42.0%) after age adjustment. In contrast, dietary recommendations were almost universal across the cohort (94.4%), with no significant differences between groups. Interestingly, overall dietary adherence was relatively high, with more than half of patients reporting compliance with dietary recommendations. Dietary records were available for 63.1% of the cohort, with a markedly lower proportion of missing records in the transition group compared with the non-transition group (17.1% vs. 43.7%). Although this difference was significant in crude analysis, it was attenuated after adjustment for age, indicating that age acted as a major confounding factor. This suggests that some of the apparent effect of the transition process may instead reflect age-related differences in patient support and engagement. This may also be attributed to the involvement of primary caregivers, such as parents or guardians, in facilitating dietary record keeping and adherence in younger individuals. Consequently, disentangling the true impact of transition interventions from age-related behavioral and social factors remains difficult, representing an inherent limitation in assessing dietary compliance. In addition, individual use of the Odimet^®^ platform was very limited, around 7% overall, and similar between groups. This low degree of engagement with the platform, combined with the reliance on patient self-reporting, may limit the robustness of adherence estimates and should be taken into account when interpreting these results. Previous studies in PKU patients have demonstrated that structured transition programs can play a decisive role in promoting dietary adherence and improving patient-reported outcomes. For example, life satisfaction scores were reported to be up to 20 points higher in patients maintaining dietary treatment compared with those who discontinued [23]. An observational study of 111 adults with PKU further highlighted the importance of structured transitional care in supporting adherence to a low-phenylalanine diet during adolescence [21]. Moreover, long-term follow-up studies have shown that over 80% of PKU patients maintained regular contact with adult care centers after structured transition, achieving stable metabolic control and therapeutic phenylalanine levels [23]. Beyond PKU, structured dietary management has also been shown to sustain metabolic control across other specific IEM, with reported control rates ranging from 78% in adult PKU patients, as assessed through dietary records in Odimet^®^, to 100% in maple syrup urine disease, pediatric PKU, and urea cycle disorders [24].

In terms of follow-up, a high proportion of patients (88.1%) maintained active follow-up in adult care. Previous studies on transition in other chronic pediatric conditions have shown that adolescence and young adulthood are particularly vulnerable periods, often associated with increased rates of missed appointments and loss of follow-up [26,27]. However, despite the younger age of patients in the transition group in our study, no significant differences were observed between groups in the number of scheduled, missed, or rescheduled appointments. Observational studies have reported that structured transition programs may increase healthcare engagement, with one study documenting a 27% rise in the median annual number of visits during the first two years after transfer to adult care [28]. In that context, the continuity of care provided by the same nutritionists before and after the implementation of pediatric and adult services contributed to consistently high adherence throughout the study period. In addition, only 6% of patients in our cohort ceased follow-up after the first adult visit, a proportion comparable to previously reported series [28]. Taken together, these findings suggest that structured transition programs may help mitigate the risk of disengagement from care that is often observed during adolescence and young adulthood, thereby preserving long-term adherence and continuity of metabolic management.

This study has several limitations. Firstly, its retrospective and single-center design restricts generalizability, precludes causal inference, and may introduce biases inherent to data collection from medical records, including the risk of missing or incomplete information. This was particularly evident in patients with an absence of dietary records, for whom unmeasured factors such as socioeconomic background, family support, or educational level may have influenced adherence and outcomes. Secondly, the relatively small sample size, a frequent challenge in studies of rare genetic disorders, likely reduced statistical power, limiting the ability to detect significant associations and constraining the scope of multivariate analyses. Some associations observed in univariate analyses lost significance after adjustment for age, underscoring the strong confounding effect of this variable. Thirdly, although the cohort size is comparable to, and in some cases larger than, those reported in previous studies, which supports a reasonable analysis of transition outcomes in rare metabolic disorders, the wide heterogeneity of IEM diagnoses and the limited sample size for each condition restricted disorder-specific interpretation, thereby limiting the ability to draw condition-specific conclusions.

## 5. Conclusions

The findings of the current study demonstrate a positive impact of transition consultations on the follow-up and self-management of young adult patients with IEM. Structured transition care was associated with better maintenance of dietary records, supported engagement with adult services, and may contribute to the continuity of treatment and follow-up. These findings should be interpreted in the context of limited follow-up and the absence of a direct assessment of metabolic control or acute clinical outcomes. Future multicenter, prospective studies are needed to evaluate long-term impact and optimize transition strategies in patients with IEM.

## Figures and Tables

**Table 1:** Demographic, clinical, treatment, and follow-up characteristics of patients with inborn errors of metabolism, stratified by transition status.

	Overall Cohort (*n* = 160)	Transition Group (*n* = 41)	Non-Transition Group (*n* = 119)	*p* Value	*p* Value Adjusted for Age
Demographic characteristics					
Sex (*n*, % women)	95 (59.4)	21 (51.2)	74 (62.2)	0.218	0.843
Current age (years)	36.2 ± 11.5	28.12 ± 9.9	38.9 ± 10.8	<0.001	-
Age at diagnosis (months)	39 (344)	1 (131)	66 (359)	0.008	-
Age at first adult visit (years)	30.4 ± 10.9	24.4 ± 9.5	32.3 ± 10.6	<0.001	-
Follow-up time (months)	31 (24)	36 (16)	29 (34)	0.364	0.440
**Diagnostic pathway**					
Screening (*n*, % patients)	72 (45.0)	27 (65.9)	45 (37.8)	0.007	0.259
Clinical presentation (*n*, % patients)	45 (28.1)	6 (14.6)	39 (32.8)		
Family history (*n*, % patients)	43 (26.9)	8 (19.5)	35 (29.4)		
**Treatment and nutritional guidance**					
Pharmacological treatment (*n*, % patients)	74 (46.3)	24 (58.5)	50 (42.0)	0.073	0.001
Dietary recommendations (*n*, % patients)	151 (94.4)	41 (100)	110 (92.4)	0.064	0.442
No dietary records (*n*, % patients)	59 (36.9)	7 (17.1)	52 (43.7)	0.007	0.064
Patient home use of Odimet^®^ (*n*, % patients)	11 (6.9)	2 (4.9)	9 (7.6)	0.730	0.563
Overall adherence in Odimet^®^ (*n*, % patients)	83 (51.9)	20 (48.8)	63 (52.9)	0.718	0.334
**Medical appointments**					
Attended visits, median [IQR]	7 [9]	6 [6]	8 [10]	0.173	0.339
Missed visits, median [IQR]	1 [1]	1 [1]	1 [1]	0.399	0.801
Rescheduled visits, median [IQR]	0 [1]	0 [1]	0 [1]	0.852	0.957

Note: Data are *n* (%), mean ± standard deviation (SD) or median [interquartile range, IQR] as appropriate.

## Data Availability

The data presented in this study are available on request from the corresponding authors due to privacy and ethical restrictions.

## Supplementary Materials

The following supporting information can be downloaded at: https://www.mdpi.com/article/10.3390/nu17203240/s1, Table S1: Detailed classification and frequency of inborn errors of metabolism in the study cohort.
