# Supplementary material for: Impact of a Transition Clinic on Long-Term Care and Nutritional Management in Patients with Inborn Errors of Metabolism
Source: Nutrients. 2025 Oct 15;17(20):3240. doi: 10.3390/nu17203240 (PMC12567094; doi:10.3390/nu17203240)
Supplement: Supplementary file 1 [file nutrients-17-03240-s001.zip › nutrients-3893672-supplementary.pdf]

Table S1. Detailed classification and frequency of inborn errors of metabolism in the study cohort.

Metabolic disorder category	n (%) patients	Specific IEM diagnosis	n (%) patients	OMIM #
Disorders of amino acid metabolism	104 (65.0)	Phenylalanine hydroxylase deficiency	59 (36.9)	261600
		Cystinuria	20 (12.5)	220100
		Glutaryl-CoA dehydrogenase deficiency	4 (2.5)	231670
		3-Methylcrotonyl-CoA carboxylase 1 deficiency	3 (1.9)	210200
		Methionine adenosyltransferase I/III deficiency	3 (1.9)	250850
		Ornithine transcarbamylase deficiency	3 (1.9)	311250
		Alpha-aminoadipic semialdehyde synthase deficiency	2 (1.3)	605113
		Cystathionine beta-synthase deficiency	2 (1.3)	236200
		Glycine N-methyltransferase deficiency	2 (1.3)	606664
		Methylmalonic aciduria	2 (1.3)	251000
		Nonketotic hyperglycinemia due to aminomethyltransferase deficiency	2 (1.3)	238310
		Fumarylacetoacetase deficiency	1 (0.6)	276700
		Homogentisic acid oxidase deficiency	1 (0.6)	203500
Disorders of carbohydrate metabolism	19 (11.9)	Fructose-1,6-bisphosphatase deficiency	6 (3.8)	229700
		Glucose-6-phosphatase deficiency	5 (3.1)	232200
		Glucose-6-phosphate transporter deficiency	3 (1.9)	602671
		Galactose-1-phosphate uridylyltransferase deficiency	2 (1.3)	230400
		Glucose transporter 1 deficiency	2 (1.3)	606777
		Phosphorylase kinase subunit beta deficiency	1 (0.6)	172490
Disorders of vitamin and cofactor metabolism	16 (10.0)	Biotinidase deficiency	10 (6.3)	253260
		Methylenetetrahydrofolate dehydrogenase 1 deficiency	6 (3.8)	172460
Disorders of fatty acid and ketone body metabolism	11 (6.9)	Carnitine palmitoyltransferase 2 deficiency	4 (2.5)	608836
		Medium-chain acyl-CoA dehydrogenase deficiency	3 (1.9)	201450
		Carnitine-acylcarnitine translocase deficiency	2 (1.3)	212138
		Short-chain acyl-CoA dehydrogenase deficiency	2 (1.3)	201470
Disorders of tetrapyrrole metabolism	3 (1.9)	Porphobilinogen deaminase deficiency	3 (1.9)	176000
Disorders of lipid metabolism	2 (1.3)	Alpha-methylacyl-CoA racemase (AMACR) deficiency	1 (0.6)	614307
		Choline kinase beta deficiency	1 (0.6)	612395
Disorders of complex molecule degradation	2 (1.3)	Iduronate sulfatase deficiency	1 (0.6)	309900
		Neutral sphingomyelinase 3 deficiency	1 (0.6)	605777
Disorders of nucleobase, nucleotide and nucleic acid metabolism	1 (0.6)	CAD trifunctional protein deficiency	1 (0.6)	114010
Disorders of energy substrate metabolism	1 (0.6)	Guanidinoacetate methyltransferase deficiency	1 (0.6)	612736
Endocrine metabolic disorders	1 (0.6)	Insulin receptor deficiency	1 (0.6)	147670

Classification of inborn errors of metabolism was based on the International Classification of Inherited Metabolic Disorders (ICIMD). IEM: inborn errors of metabolism; CAD: Carbamoyl-phosphate synthetase 2, Aspartate transcarbamylase, and Dihydroorotase.
